# Dual PI3K/Akt Inhibitors Bearing Coumarin-Thiazolidine Pharmacophores as Potential Apoptosis Inducers in MCF-7 Cells

**DOI:** 10.3390/ph15040428

**Published:** 2022-03-31

**Authors:** Rana M. Abdelnaby, Heba S. Rateb, Omaima Ali, Ahmed S. Saad, Rania I. Nadeem, Sahar M. Abou-Seri, Kamilia M. Amin, Nancy S. Younis, Rasha Abdelhady

**Affiliations:** 1 Pharmaceutical Chemistry Department, Faculty of Pharmacy, Heliopolis University, Cairo 11785, Egypt; 2 Pharmaceutical Chemistry Department, Faculty of Pharmaceutical Science and Drug Manufacturing, Misr University for Science and Technology, 6th of October City 12585, Egypt; heba.sayed@must.edu.eg; 3 Egyptian Drug Authority, Cairo 12618, Egypt; omaima_salah@hotmail.com; 4 Pharmacology and Toxicology Department, Faculty of Pharmacy, Port Said University, Port Said 42511, Egypt; mosa1200@yahoo.com; 5 Pharmacology and Toxicology Department, Faculty of Pharmacy, Heliopolis University, Cairo 11785, Egypt; rania.ibrahim@hu.edu.eg; 6 Pharmaceutical Chemistry Department, Faculty of Pharmacy, Cairo University, Cairo 11562, Egypt; sahar.shaarawy@pharma.cu.edu.eg (S.M.A.-S.); kamilia.amin@pharma.cu.edu.eg (K.M.A.); 7 Pharmaceutical Sciences Department, Faculty of Clinical Pharmacy, King Faisal University, Al Hofuf 31982, Al-Ahsa, Saudi Arabia; nyounis@kfu.edu.sa; 8 Pharmacology and Toxicology Department, Faculty of Pharmacy, Fayoum University, Fayoum 63514, Egypt; ram14@fayoum.edu.eg

**Keywords:** PI3K/Akt pathway, MCF-7, 7-hydroxycoumarin, thiazolidin-4-ones, apoptosis, anticancer activity

## Abstract

Breast cancer is the most common malignancy worldwide; therefore, the development of new anticancer agents is essential for improved tumor control. By adopting the pharmacophore hybridization approach, two series of 7-hydroxyl-4-methylcoumarin hybridized with thiosemicarbazone (**V–VI**) and thiazolidin-4-one moieties (**VII–VIII**) were prepared. The in vitro anticancer activity was assessed against MCF-7 cells adopting the MTT assay. Nine compounds showed significant cytotoxicity. The most promising compound, **VIIb**, induced remarkable cytotoxicity (IC_50_ of 1.03 + 0.05 µM). Further investigations were conducted to explore its pro-apoptotic activity demonstrating S-phase cell cycle arrest. Apoptosis rates following **VIIb** treatment revealed a 5-fold and 100-fold increase in early and late apoptotic cells, correspondingly. Moreover, our results showed caspase-9 dependent apoptosis induction as manifested by an 8-fold increase in caspase-9 level following **VIIb** treatment. Mechanistically, **VIIb** was found to target the PI3K-α/Akt-1 axis, as evidenced by enzyme inhibition assay results reporting significant inhibition of examined enzymes. These findings were confirmed by Western blot results indicating the ability of **VIIb** to repress levels of Cyclin D1, p-PI3K, and p-Akt. Furthermore, docking studies showed that **VIIb** has a binding affinity with the PI3K binding site higher than the original ligands X6K. Our results suggest that **VIIb** has pharmacological potential as a promising anti-cancer compound by the inhibition of the PI3K/Akt axis.

## 1. Introduction

Cancer is a multifactorial disease that ranks as the second leading cause of death globally, causing approximately 10 million deaths in 2020. Notably, female breast cancer was reported as the most frequently diagnosed cancer type, surpassing lung cancer [1,2]. The incidence of breast cancer varies globally, it affects 1 out of 20 females worldwide and 1 out of 8 in developing countries [3]. Major risk factors for breast cancer occurrence include both modifiable factors, such as lifestyle, diet, or hormone replacement therapy, and non-modifiable factors, such as age, sex, race, and genetic makeup [4].

Although surgery and chemotherapy are the mainstream therapeutic strategies for breast cancer, still the development of novel targeted cancer therapies sparing toxicities to off-cancer cells is an urgent need, mainly due to the major limitations of conventional chemotherapeutic agents, including systemic toxicity and multidrug resistance [3,5].

It has been noted that several signaling pathways are dysregulated and subsequently have been implicated in the pathogenesis of breast cancer. Remarkably, the signaling pathway defined by the phosphatidylinositol-3-kinase (PI3K)/protein kinase B (Akt) axis is a chief controller of a myriad of cellular functions, including cell growth and proliferation. Moreover, aberrations in this molecular pathway are critical in breast tumor initiation, survival, and angiogenesis [5,6,7,8]. Notably, oncogenic activation of this pathway in breast cancer is mainly attributed to the mutation of genes encoding PI3K subunits, including p110α (PIK3CA) and p110β (PIK3CB), where PIK3CA mutations were reported in 30–40% of breast cancer patients [9,10].

**Figure 1:**
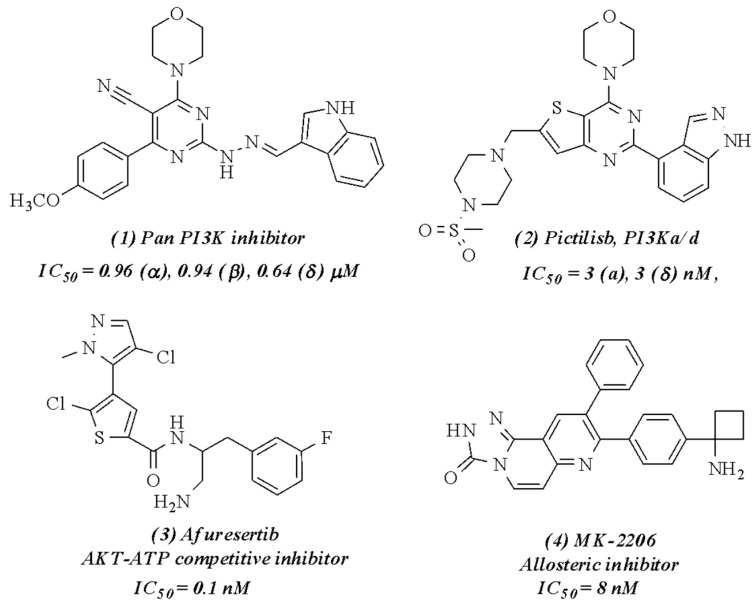
Reported PI3K and Akt inhibitors. Structures 1 and 2 are adapted from [17], 3 from [18], and 4 from [19].

Furthermore, the role of the PI3K/Akt signaling network in cancer cells immunomodulation has been clearly highlighted, since Akt hyperactivation was associated with the escape of cancer cells from immune recognition [11]. Consequently, recent studies documented that inhibition of the PI3K/Akt axis enhances tumor immunosurveillance by inhibiting the activation of immunosuppressive pathways [5,6,12]. Moreover, in breast cancer, dysregulation of this signaling axis plays a principal role in resistance to antineoplastic chemotherapeutic drugs, hormonal therapy, and targeted therapy [5,7,13]. Lately, the pivotal role of the PI3K/Akt axis in breast carcinogenesis has been characterized, prompting the development of recent therapeutic strategies that could inhibit this pathway aiming at both limiting tumor proliferation and/or survival as well as reviving tumor functional immunosurveillance. Many clinical studies have proved that inhibitors acting on different enzymes of this pathway (Figure 1) are very successful therapeutic agents with benefits against the emergence of resistance and with better disease prognosis [14,15,16].

**Figure 2:**
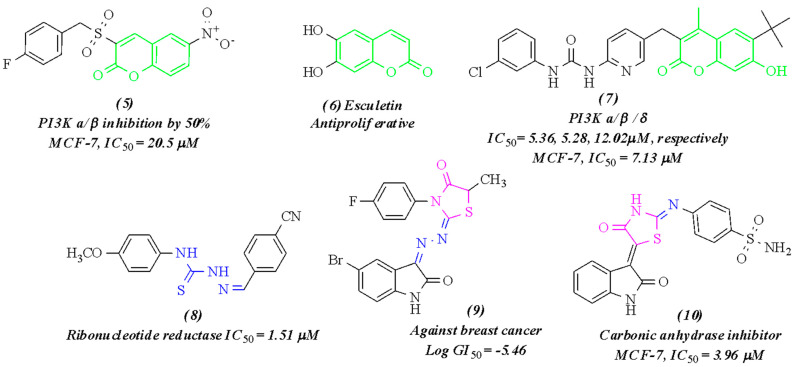
Reported coumarin and thiazolidine containing anticancer agents. Structures 5 to 10 are adapted, respectively, from [20,24,27,28,31,32].

In the war on cancer, natural products and their derivatives have played a crucial role in developing effective chemotherapeutic agents such as vinca alkaloids and taxols, which inspired our research team to develop new chemotherapeutic agents adopting naturally found scaffolds, such as a coumarin ring that was reported over the years to have potent anticancer activity through manipulation of many cellular mechanisms (Figure 2) [20,21,22,23,24].

**Figure 3:**
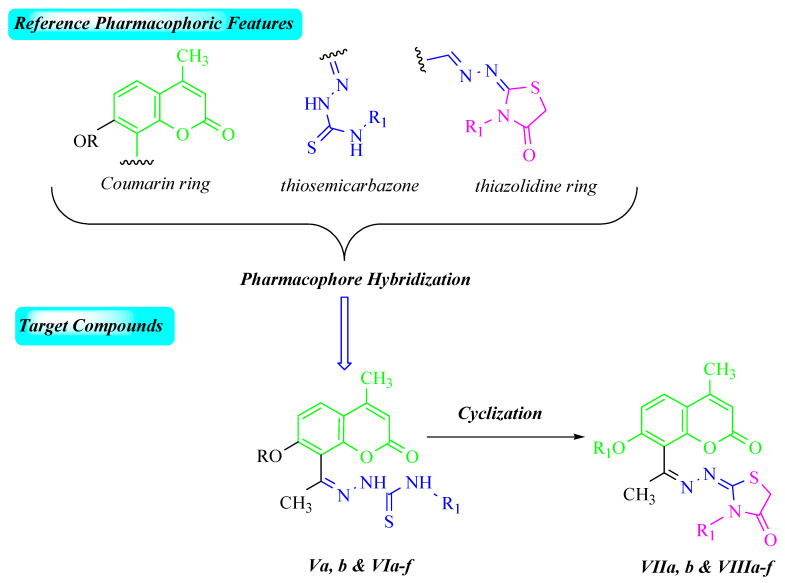
Design of the new compounds (**V–VIII**).

The molecular hybridization strategy has emerged as a novel approach that involves combining two or more pharmacophores in one molecule with the benefits of having a better pharmacological profile in either additive (acting on the same biological target) or synergistic (modulating different targets) manner for the parent molecules and less likely to develop drug resistance. That attracted large groups of researchers to investigate different scaffolds in chemotherapeutic agents separately and their merging in one molecule [25,26]. Literature survey revealed the potent anticancer effect of several coumarin derivatives (Figure 2) owing to their multi-targeting mechanism of action in cell biology, for example, apoptosis induction and PI3K/AKT inhibition, which all finally stop cell proliferation and survival processes [20,21,22,23,24]. Also, compounds featuring thiosemicarbazone linker and its cyclic analog thaizolidin-4-one ring showed promising effectiveness against many cancer types as result of ribonucleotide reductase, and carbonic anhydrase inhibition, (Figure 2) [27,28,29,30]. Hence, the target compounds (**V–VIII**) were designed adopting the pharmacophore hybridization technique to have a coumarin scaffold as the main nucleus merged with thiosemicarbazone linker or thaizolidin-4-one ring at position C8 of the coumarin ring, as represented in Figure 3.

This research work aimed at evaluating the potential cytotoxicity of the novel derivatives in breast cancer cell line MCF-7. Further studies were conducted for the most promising compound, exploring the enzyme inhibition assay, examining PI3K-α, PI3K-γ, and Akt-1 isoforms. Secondly, the proapoptotic activity was assessed via investigating cell cycle distribution alongside apoptosis percentage. In addition, the downstream proteins of the signaling pathway under investigation were evaluated by Western blot. Eventually, examination of the binding interactions between the promising derivatives and the nominated enzymes was conducted by molecular modeling.

## 2. Results and Discussion

### 2.1. Chemistry

**Scheme 1:**
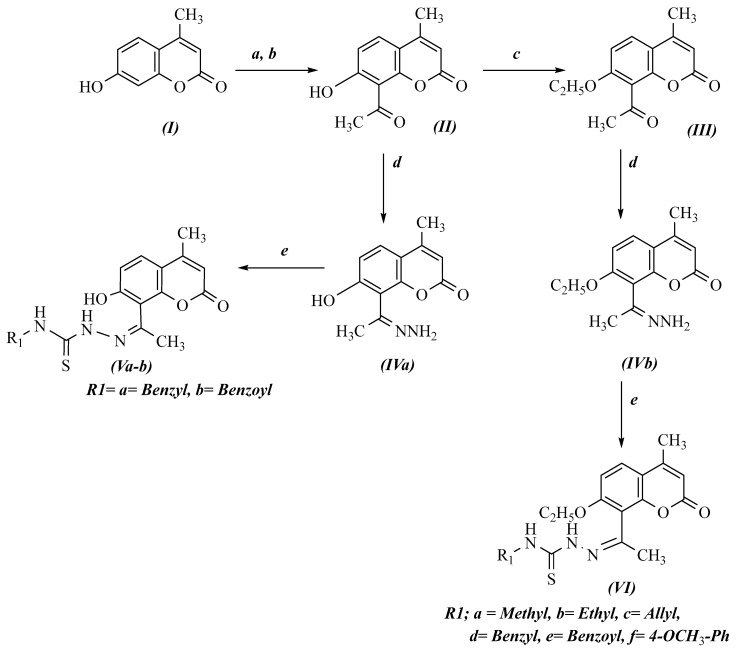
Reagents and conditions: (**a**) Ac_2_O; (**b**) AlCl_3_, fusion; (**c**) ethyl bromide, K_2_CO_3_/dry acetone; (**d**) N_2_H_4_·H_2_O, ethanol; (**e**) isothiocyanate derivatives, ethanol.

**Scheme 2:**
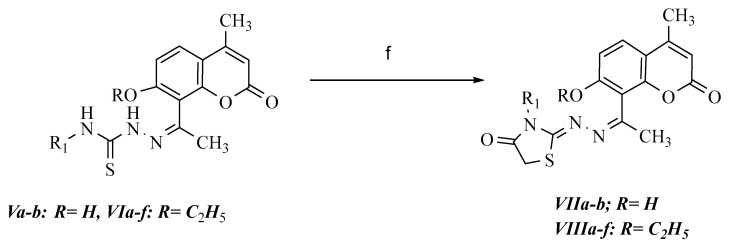
Reagents and conditions: (**f**) chloroacetic acid, NaAc/ethanol.

As depicted in Scheme 1 and Scheme 2, the designed compounds were prepared. Subsequently, structure confirmation was done using spectral data and elemental analysis described in the materials and methods section. The starting compounds, **I–IV**, were prepared as reported by our research team in a previous study [33]. Coumarin-thiosemicarbazones (**Va**, **Vb**, and **VIa–f**) resulted from the reaction of the hydrazone intermediates **IVa**, b with different isothiocyanates in good yield; FT-IR exhibited the disappearance of primary amine peak and only showed two secondary amine peaks at 3200–3400 cm^−1^ [34]. Moreover, ^1^H-NMR showed extra aromatic protons at 7.3–7.6 ppm for **Va** and **Vb** analogs, while for the **VIa-f**, there were the corresponding peaks for the aliphatic substitution at 1.33 and 2.4 ppm for **VIa** and **VIb**, 4–6 ppm for the allyl group in **VIc**, and the extra aromatic protons at 7.0–7.7 ppm for **VIIf**, **g**, **h** derivatives. Coumarin-thiazolidine-4-ones (**VII–VIII**) then were synthesized via the cyclization reaction with chloroacetic acid; the FT-IR showed the disappearance of amino peaks and the appearance of a second carbonyl peak at 1700 cm^−1^, while the ^1^H-NMR presented a peak at δ = 2.85–3.86 ppm of the methylene group in the thiazolidine ring [35].

### 2.2. Antitumor Activity

#### 2.2.1. Cytotoxicity Assay

The in vitro anticancer activity of the designed coumarin-thiosemicarbazones and coumarin-thiazolidine-4-one hybrids against MCF-7 cells was tested using the MTT assay, and 5-fluorouracil (5-FU) was used as the reference drug. The tested compounds were used in different concentrations, and cell survival was determined after incubation for 48 h as reported [36,37]. The cytotoxic activity is represented in Table 1 as IC_50_ (µM) values.

The cytotoxicity assay results highlighted that both series (open and cyclized analogs) are promising candidates as antitumor agents ranging from highly active to moderate activity with IC_50_ of 1.03–79.90 µM.

In the coumarin-thiosemicarbazone series (**Va**,**b**, and **VIa–f**), the different aliphatic and aromatic substitutions showed promising cytotoxicity, where some compounds demonstrated IC_50_ values statistically significantly lower than that of the reference compound, 5-FU (IC_50_ = 27.81 + 1.41 µM). Notably, when R = H, the benzyl analog (**Va**) gave promising inhibition with IC_50_ = 5.13 + 0.28 µM, while the benzoyl derivative showed lower activity with IC_50_ = 47.32 µM.

Moreover, when R = C_2_H_5_, the reported results varied between the aliphatic and aromatic substitutions. The benzyl derivative (**VId**) showed better activity than its 7-hydroxyl analog (**Va**), while for the benzoyl derivative (**VIe**), the activity did not get any better but the methoxy derivative showed highly potent inhibition with IC_50_ = 1.21 µM. For the aliphatic substitutions, the methyl (**VIa**) and ethyl (**VIb**) derivatives showed good to moderate activities with IC_50_ of 11.13 and 38.70 µM, while the allyl derivative **VIc** showed highly potent activity with IC_50_ = 2.72 µM.

By looking at the cyclic analogs, thiazolidin-4-one series, the 7-hydroxy coumarin analogs (**VIIa**, **b**), the cyclization led to decreased activity for the benzyl derivative **VIIa** (IC_50_ = 54.80 µM), while the benzoyl derivatives **VIIb** (IC_50_ = 1.03 µM) gave the best activity compared to the open analog **Vb** (IC_50_ = 47.32 µM).

In the 7-ethoxy series (**VIIIa-f**), the compounds had lower inhibitory activity than the open analogs. The derivatives that displayed higher cytotoxicity than the reference compound, 5-FU, were **VIIIa**, **VIIIc**, and **VIIIf**, showing IC_50_ values of 20.27, 4.95, and 11.80 µM, respectively, while **VIIIe** (IC_50_ = 26.04 µM) gave comparable activity to 5-FU but better than the open analog that resulted in IC_50_ = 43.05 µM.

However, IC_50_ values recorded for Compounds **Vb**, **VIb**, **VIe**, **VIIa**, **VIIIb**, and **VIIId** were 47.32, 38.70, 43.05, 54.8, 57.28, and 79.93 µM, correspondingly, which were higher than the reported value for the reference compound 5-FU.

Statistical significance was tested using an independent t-test comparing the recorded IC_50_ values (µM) for the compounds that showed higher cytotoxicity than the reference compound to that of 5-FU where it revealed statistically significant differences for Compounds **Va**, **VIa**, **VIc, VId**, **VIf**, **VIIb**, **VIIIc**, and **VIIIf** (*p*-values < 0.001) as well as Compound **VIIIa**, (*p*-value < 0.01) vs. the reference compound 5-FU. Moreover, the IC_50_ of Compound **VIIIe** was non-significantly different than that of 5-FU (*p*-value = 0.27).

The metabolic viability of the most potent compounds, **VIf** and **VIIb**, in non-cancerous epithelial cells (MCF 10), was examined using an MTT assay calculating the selectivity index (SI) [38]. The data provided in Table 2 illustrated that both **VIf** (IC_50_ = 20.11 ± 1.05, SI = 16.61) and **VIIb** (IC_50_ = 9.52 ± 0.60, SI = 9.24) have a promising safety profile compared to 5-FU (IC_50_ = 36.22 ± 1.89, SI = 1.30). Herein, such results suggested that at the selected doses, **VIf** and **VIIb** potentially will not cause deleterious effects to neighboring non-cancer cells.

Furthermore, the findings of the MTT test proved that the hybridization technique adopted between coumarin and thiosemicarbazone or thiazolidine-4-one gave highly active compounds, rendering them very promising candidates for further investigations. The current study focused on investigating the potential anticancer mechanism for the novel compound **VIIb** in MCF-7 cells, whilst the mechanistic details for compound **VIf** will be explored in our future work to ponder its anticancer effects in a different cancer cell line.

#### 2.2.2. Enzyme Inhibition Assay

Accumulating evidence highlighted that PI3K signaling pathway upregulation is highly implicated in breast cancer development and disease progression as well as resistance to hormones and cytotoxic therapy. Therefore, it is essential to elucidate the effect of the novel compound, **VIIb**, on the PI3K/Akt signaling pathway by using in vitro PI3K class 1A enzyme inhibition assay conducted on PI3K-α and PI3K-γ isoforms as well as an Akt-1 enzyme inhibition assay. Notably, PI3K-α is the most mutated isoform in the PI3K pathway in breast cancer, whilst the PI3K-γ isoform is not commonly mutated in breast cancer. Furthermore, targeting the PI3K-γ isoform in breast cancer could contribute to enhancing the anti-tumor immunity [39].

Data shown in Table 3 demonstrated that **VIIb** treatment displayed a potent inhibitory activity on PI3K-α and Akt-1 isoforms where the reported IC_50_ values were statistically significantly lower than the reference compound, LY294002. The IC_50_ values were found to be 3.70 ± 0.19 and 2.93 ± 0.15 μM, for PI3K-α and Akt-1, correspondingly. Furthermore, **VIIb** treatment displayed PI3K-γ isoform inhibition with IC_50_ of 34.70 ± 1.88 µM. However, the recorded IC_50_ value was higher than the reported value of the reference drug.

These findings proved that the hybridization technique adopted in this work succeeded in generating a promising dual inhibitor for the PI3K/Akt axis, which could be beneficial in treating either hormone- or chemo-resistant breast cancers.

#### 2.2.3. Cell Cycle Analysis and Apoptosis Induction

**VIIb**-treated cells were subjected to cell cycle phase distribution analysis as well as apoptosis rates determination by flow cytometric analysis in MCF-7 cells, as reported [40,41].

**Figure 4:**
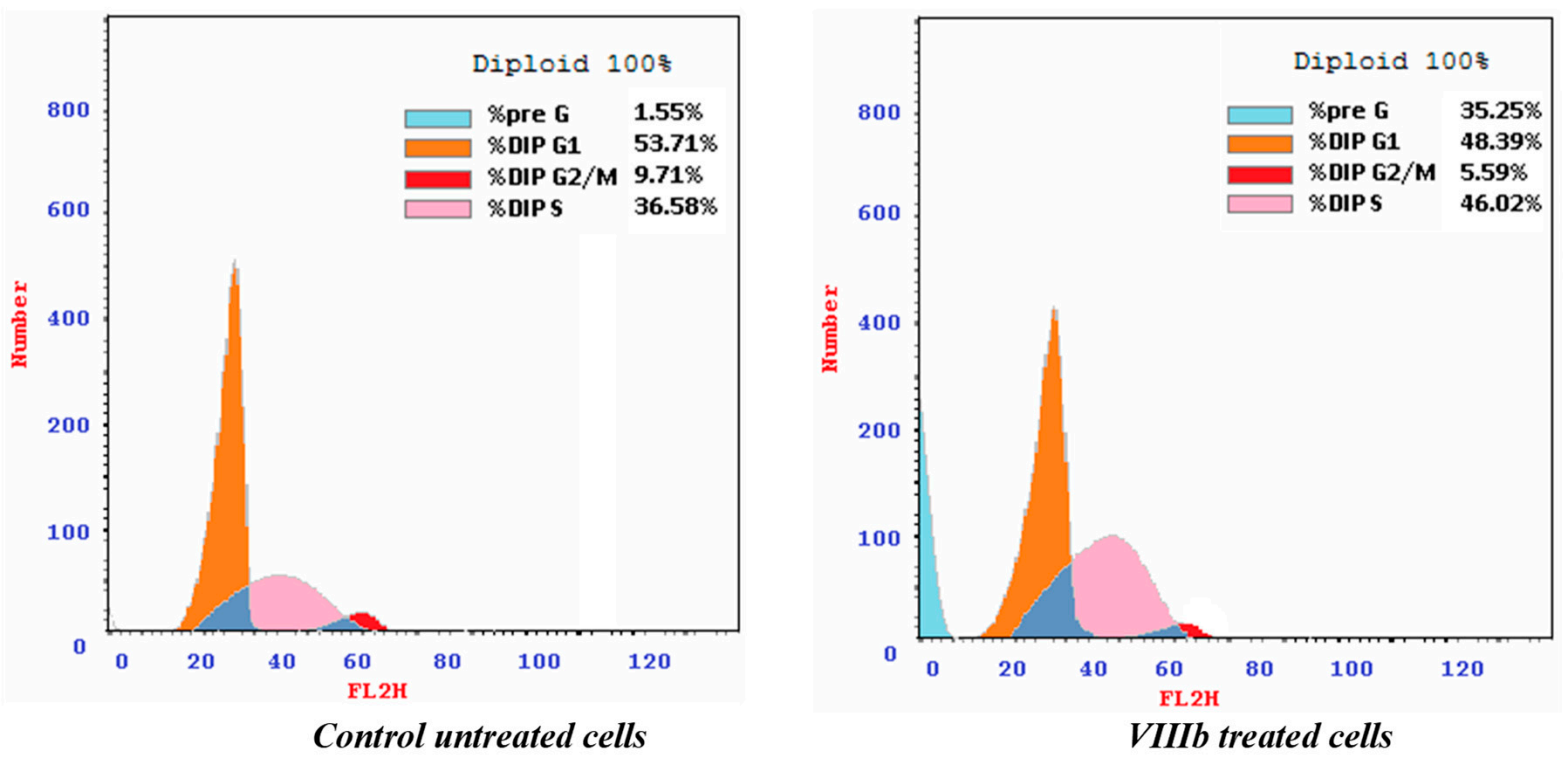
The cell distribution in cell cycle phases following Compound **VIIb** exposure.

The results of cell cycle analysis revealed marked variability between **VIIb**-exposed cells vs. control untreated MCF-7 cells, as shown in Table 4 and Figure 4, where **VIIb**-treated cells showed a higher S-phase population of 46.02% compared to 36.58% in control cells.

However, **VIIb** treatment suppressed both G0/G1 and G2/M proportions from 53.71% and 9.71%, respectively, in control untreated MCF-7 cells, to 48.39% and 5.59% in **VIIb**-exposed cells, whereas pre-G1 cells, representing apoptotic cells, had a low proportion and reached 1.55% in untreated cells that significantly increased to 35.25% following **VIIb** treatment. These data suggested that **VIIb** treatment induced S-phase accumulation and thereby S-phase arrest and potentially subsequently cell death. Results of the current study accord well with earlier research that highlighted the ability of coumarins to induce arrest of various cell cycle phases, potentially leading to apoptosis [26].

Control and **VIIb**-treated (IC_50_ µM, 48 h) MCF-7 cells were harvested and then subjected to cell cycle analysis by flow cytometry.

Remarkably, clinical oncology aims at developing novel targeted cancer therapies that could induce apoptosis in neoplastic cells to enhance their eradication. Since loss of apoptosis is closely related to cancer cell survival and abnormal growth, induction of apoptotic signaling pathways is a crucial mechanism in targeted cancer therapy. To further explore the link between apoptosis rates and **VIIb** treatment, the pro-apoptotic activity of this novel compound was investigated by flow cytometry using both Annexin V (V) and propidium iodide (PI) dyes. A distinctive feature of early apoptosis is phosphatidylserine transfer toward the cellular surface. Thus, phosphatidylserine can be detected by fluorochrome-tagged anticoagulant protein V. Therefore, the viable cells remained unstained (V^−^/PI^−^). Furthermore, early apoptotic cells were stained with V but not PI, demonstrating (V^+^/PI^−^) staining. However, late apoptotic cells showed V positive/PI positive (V^+^/PI^+^) staining, indicating the loss of integrity of the nuclear or plasma membrane [42,43,44,45].

**Figure 5:**
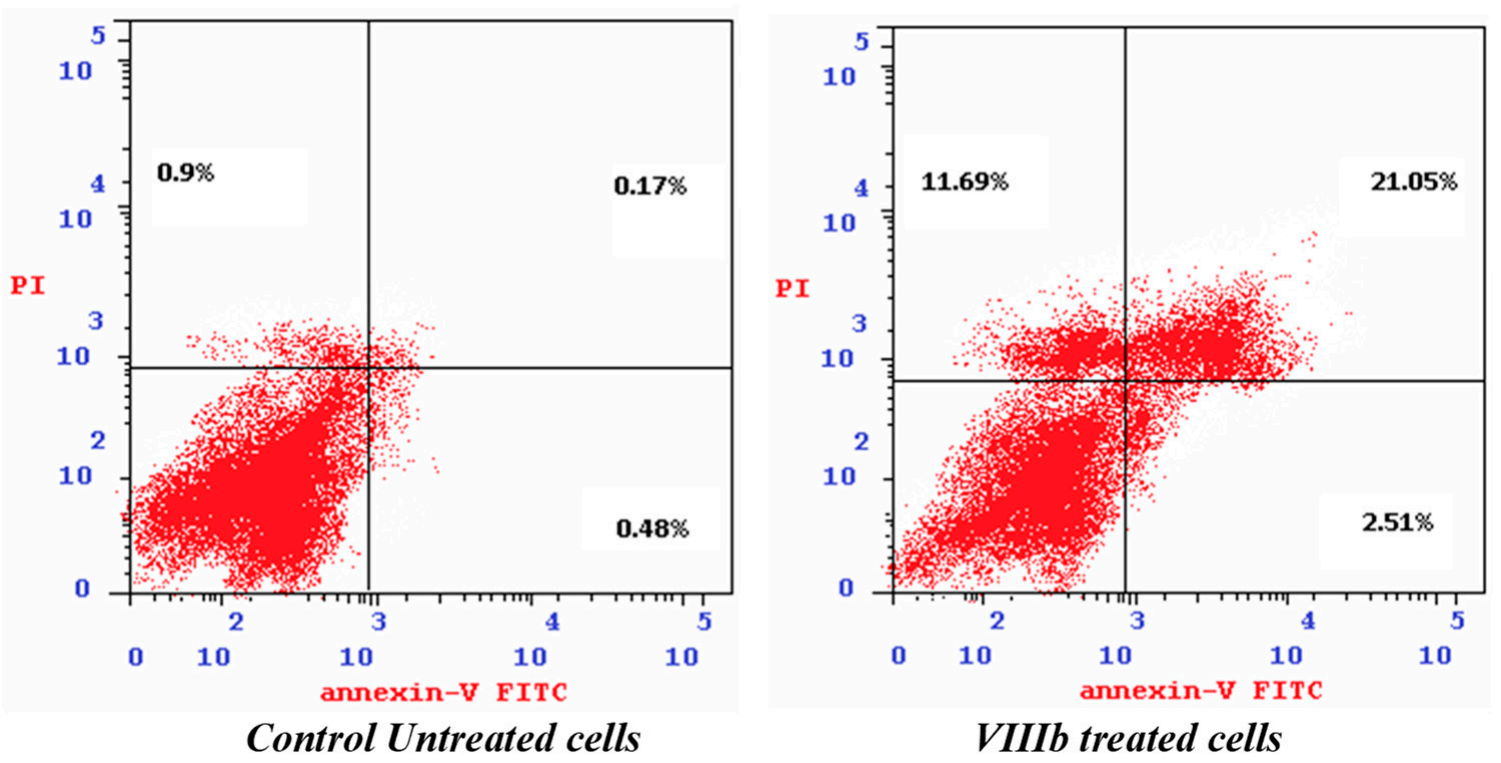
Effect of Compound **VIIb** on apoptosis induction in MCF-7 cell line.

Data of the current study highlighted that in non-treated MCF-7 cells, 0.48% and 0.17% of examined cells demonstrated V^+^/PI^−^ and V^+^/PI^+^ staining patterns, correspondingly, as shown in Table 5 and Figure 5. Interestingly, treatment of MCF-7 cells with **VIIb** caused 5-fold and 100-fold increases in early and late apoptotic cells, respectively, with respect to control cells where 2.51% and 21.05% of the cells exhibited V^+^/PI^−^ and V^+^/PI^+^ staining patterns, correspondingly.

Control untreated and MCF-7 cells treated with compound **VIIb** at its IC_50_ (µM) for 48 h were subjected to apoptotic analysis using Annexin V (V) and propidium iodide (PI) fluorescent dyes.

Results of cell cycle analysis alongside apoptosis induction highlighted the pro-apoptotic activity of the investigated compound (**VIIb**). Furthermore, the reported increase in the necrotic cells following **VIIb** treatment could be assigned to the hydroxyl group in the structure. In addition, this study demonstrated that **VIIb** exposure induced changes in the cellular distribution at different cell cycle phases.

Notably, **VIIb** exposure resulted in S-phase cell cycle arrest accompanied by a reduction in the percentage of cells in other phases. The observed effect is possibly attributed to the down-regulation of the G1-S checkpoint gene P21 that could have allowed G1-S cell cycle transition despite the presence of DNA damage [46,47]. Moreover, the reported S-phase cell cycle arrest could be mediated through cyclin A2, which is a pivotal regulator of the cell cycle and crucial for S-phase and mitotic entry [40,41]. These hypotheses will be investigated in our future work.

#### 2.2.4. Caspase-9 Assay

Apoptosis is a programmed cell death modality that includes an array of steps such as activation of caspases alongside endonucleases leading to DNA cleavage, eventually causing the formation of apoptotic bodies. Caspases are classified into effector caspases, such as caspase-3, -6, and -7, and initiator caspases, including either caspase-2, -8, and -10 (extrinsic pathway) or caspase-9 (intrinsic pathway) [48]. The level of cleaved caspase-9 was investigated in response to **VIIb** treatment as an indicator of induction of the apoptotic pathways, where our results revealed that exposure of MCF-7 cells to Compound **VIIb**, for 48 h, induced an approximately 8-fold increase in the cleaved form of caspase-9 (*p* < 0.001) (Table 6). This result supports our initial hypothesis that the potential anticancer mechanism of **VIIb** was through induction of apoptotic pathways.

Data of the current study accord well with a previous report that stated the induction of apoptosis by a novel coumarin–chalcone hybrid via activation of initiator caspase-9 [49]. Moreover, an earlier study previously highlighted that the anticancer activity of coumarin derivatives was through induction of caspase-dependent apoptotic pathways [24].

#### 2.2.5. Western Blot

**Figure 6:**
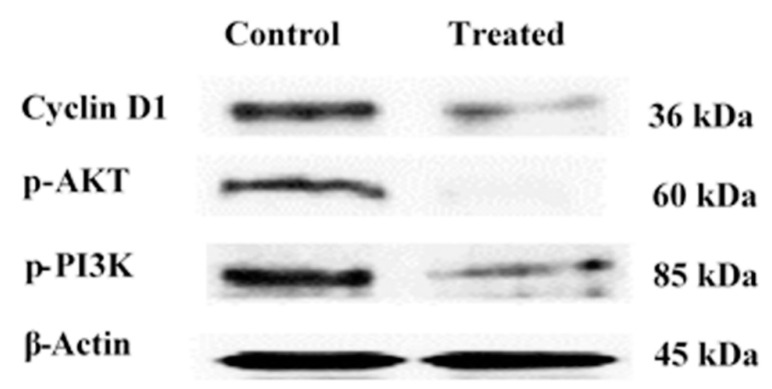
Effect of **VIIb** on Cyclin D1, p-Akt, and p-PI3K protein levels.

Cyclin D1 activity is essential for the G1 to S phase transition. Moreover, the PI3K/Akt axis is crucial for cell growth and apoptosis. The effects of **VIIb** on Cyclin D1 and the PI3K/Akt pathway in MCF-7 cells was investigated adopting reported procedures [50,51]. As shown in Figure 6, following compound **VIIb** exposure, the levels of Cyclin D1, p-PI3K, and p-Akt in MCF-7 cells were effectively suppressed with respect to control untreated cells. Herein, Western blot results confirmed our previous findings, highlighting the ability of the novel compound, **VIIb**, to inhibit the PI3K/Akt signaling pathway. Our results accord with an earlier study demonstrating that coumarin compounds suppressed Cyclin D1 and p-Akt protein levels [51]. Consequently, the current work demonstrated that **VIIb** exposure induced inhibition of Cyclin D1, which could be attributed to the reported PI3K inhibition by **VIIb**, as previously stated [52,53]. Cyclin D1 is a proto-oncogene and a major regulator of cell cycle progression. In addition, Cyclin D1 overexpression has been linked to both breast tumorigenesis and tumor progression as well as to the development of endocrine resistance in breast cancer cells [54,55]. Thus, the illustrated downregulation of Cyclin D1 following **VIIb** treatment suggests that the novel compound, **VIIb**, has pharmacological potential as a therapeutic agent capable of targeting human breast cancer.

Whole-cell protein lysates obtained from both control as well as **VIIb**-treated (IC_50_ (µM), for 48 h) MCF-7 cells were resolved by SDS-PAGE, and then immunoblotting was conducted with antibodies against cyclin D, p-Akt, and p-PI3K compared to the housekeeping protein β-Actin.

#### 2.2.6. In Silico Molecular Simulations

**Figure 7:**
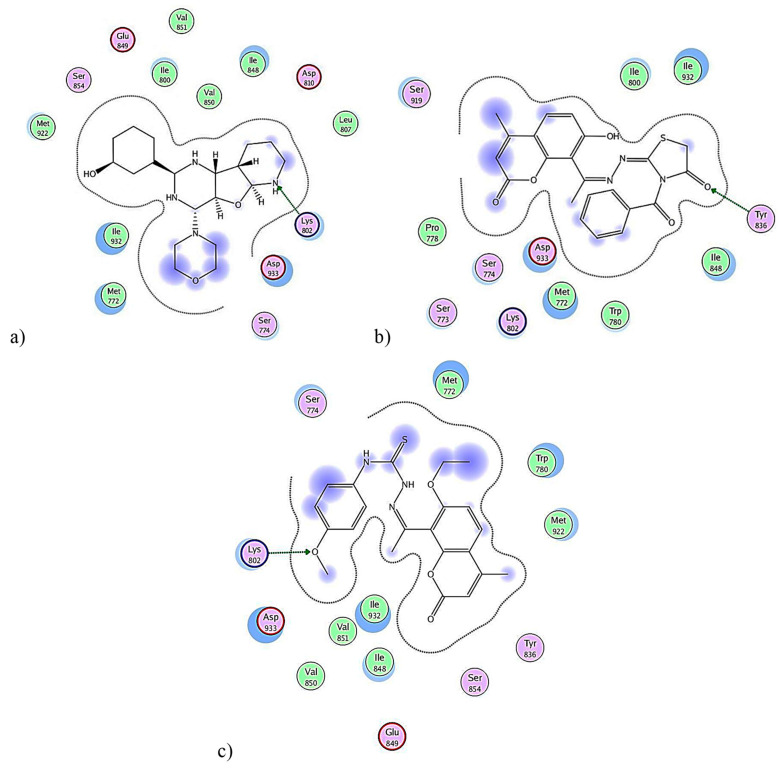
The 2D binding pattern of compounds: (**a**) **X6K** reference ligand; (**b**) **VIIb**; (**c**) **VIf** in the active site of PI3K.

**Figure 8:**
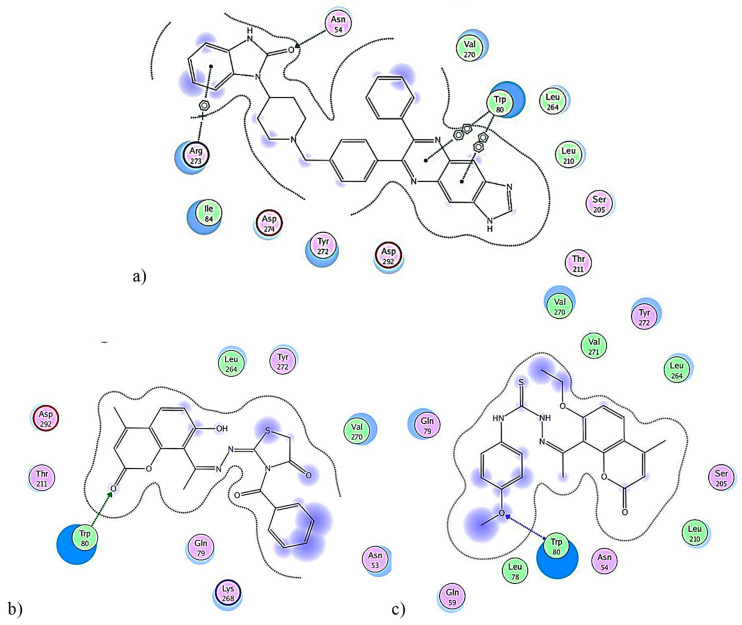
The 2D binding patterns of compounds: (**a**) **IQO** reference ligand; (**b**) **VIIb**; (**c**) **VIf** in the active site of Akt.

The integration between experimental and computational methods is a highly attractive methodology in the design and optimization field of different drug candidates. Accordingly, molecular docking was conducted to illustrate the binding interactions of the novel derivatives **VIIb** and **VIf** inside the active site of PI3K-α and Akt-1. It was reported that both Lys 802 and Tyr 836 have a pivotal role in the binding of PI3K (PDB ID. 4L23) to its inhibitors [56], whereas the most important amino acid residues in Akt-1 (PDB ID. 3O96) are Asn54, Trp80, Ser205, Glu267, Lys268, Asn269, and Arg273 [57]. The docking revealed that both PI3K and Akt-1 enzymes have good docking scores and binding affinities with both tested compounds, as shown in Table 7 and Table 8 and Figure 7 and Figure 8. The binding pattern of the two active derivatives **VIIb** and **VIf** is similar to the binding pattern of the reference ligands X6K and IQO.

## 3. Materials and Methods

### 3.1. Chemistry

All the chemical reagents were available from Sigma-Aldrich (St. Louis, MO USA). FT-IR spectral analyses (KBr discs) were conducted on a Shimadzu IR Affinity-1 spectrophotometer. ^1^H-NMR spectra were conducted on a JEOL ECA 300, 500 MHz spectrometer using CDCl_3_ as stated.

The coumarin analogs **Va**, **Vb**, **VIf**, **VIIa**, **VIIb**, and **VIIIf** were previously reported by our research group, and thiosemicarbazone derivatives **VIa-f** followed the same reported procedures [33].

#### General Procedures for Thiosemicarbazones **V** and **VI** Synthesis

The appropriate isothiocyanates (0.05 mol) were added to the hydrazone intermediates (0.05 mol), and the reaction mixture was dissolved in ethanol/dimethylformamide and reflux continued for 8 h. The solvent was removed under vacuum, and the formed solid was collected and recrystallized from ethanol.

(*Z/E*)-1(1-(7-ethoxy-4-methylcoumarin)ethylidene)-4-methyl-thiosemicarbazone (**VIa**):

Yield = 85.06%; m.p. = 202–204 °C; FT-IR (ṽ max, cm^−1^): 3286 and 3417 (2NH), 3080 (CH, Ar), 2981 (CH, aliphatic), 1732 (C=O), 1627 (C=N, imine), 1597 (C=C, Ar), and 1188 (C-O, ether); ^1^H-NMR (300 MHz, CDCl_3_) δ (ppm): 1.41 (t, j = 9 Hz, 3H, CH_3_-CH_2_-O), 2.22 (s, 3H, N=C-CH_3_), 2.27 (s, 3H, C4-CH_3_), 2.41 (d, j = 6 Hz, 3H, NH-CH_3_), 4.16–4.20 (m, 2H, CH_3_-CH_2_-O), 6.17 (s, 1H, C3-Hof coumarin), 6.92 (d, j = 9 Hz, 1H, C6-H_arom_), 7.62 (d, j = 9 Hz, 1H, C5-H_arom_), 2.8 and 8.17 (s, 2H, 2NH; exchangeable with D_2_O); ^13^C-NMR (400 MHz, CDCl_3_) δ (ppm) = 14.55 (CH_3_-CH_2_-), 18.47 (CH_3_-), 23.53 (C=N-CH_3_), 31.08 (CH_3_-NH-), 65.01 (CH_3_-CH_2_-), 108.47–114.44 (3C of coumarin), 127.37 (C5 of coumarin), 142.81 (C10 of coumarin), 151.08 (C4-CH_3_ of coumarin), 152.17 (C-O-C_2_H_5_), 157.61 (-C=O of coumarin), 159.91 (-C=N-), 178.36 (-C=S);

M+(*m*/*z*): 333; Anal calcd: C, 57.64; H, 5.74; N, 12.60; found: C, 57.82; H, 5.79; N, 12.88.

(*Z/E*)-1(1-(7-ethoxy-4-methylcoumarin)ethylidene)-4-ethyl-thiosemicarbazone (**VIb**):

Yield = 83.95%; m.p. = 183 °C; FT-IR (ṽ max, cm^−1^): 3441 and 3292 (2NH), 3080 (CH, Ar), 2981 (aliphatic CH), 1732 (C=O), 1598 (C=C, Ar), and 1184 (C-O, ether); ^1^H-NMR (400 MHz, CDCl_3_) δ (ppm): 1.33 (t, 3H, j = 8 Hz, CH_3_-CH_2_-NH), 1.42–1.44 (t, j = 8 Hz, 3H, CH_3_-CH_2_-O), 2.22 (s, 3H, N=C-CH_3_), 2.30 (s, 3H, C4-CH_3_), 3.71–3.77 (m, j = 4 Hz, 2H, NH-CH_2_-CH_3_), 4.15–4.17 (m, j = 4 Hz, 2H, CH_3_-CH_2_-O), 6.19 (s, 1H, C3-H of coumarin), 6.93 (d, j = 8 Hz, 1H, C6-H_arom_), 7.64 (d, j = 8 Hz, 1H, C5-H_arom_,), 8.10 and 8.64 (s, 2H, 2NH; exchangeable with D_2_O); M+(*m*/*z*): 347; Anal calcd: C, 58.77; H, 6.09; N, 12.09; found: C, 58.99; H, 6.18; N, 12.41.

(*Z/E*)-1(1-(7-ethoxy-4-methylcoumarin)ethylidene)-4-allyl-thiosemicarbazone (**VIc**):

Yield = 97.7%; m.p. = 100–102 °C; FT-IR (ṽ max, cm^−1^): 3381 and 3429 (2NH), 3080 (CH, Ar), 2980 (aliphatic CH), 1732 (C=O), and 1597 (C=C, Ar, allyl); ^1^H-NMR (300 MHz, CDCl_3_) δ (ppm): 1.40 (t, j = 6 Hz, 3H, CH_3_-CH_2_-O), 1.74 (s, 3H, N=C-CH_3_), 2.29 (s, 3H, 4-CH_3_), 4.18 (q, j = 6 Hz, 2H, CH_3_-CH_2_-O), 4.3 (d, j = 9 Hz, 2H, NH-CH_2_-CH=CH_2_), 5.22 (d, j = 9 Hz, 2H, CH_2_-CH=CH_2_), 6.00 (m, 1H, CH_2_-CH=CH_2_), 6.16 (s, 1H, C3-H_of_ coumarin), 6.93 (d, j = 9 Hz, 1H, C6-H_arom_), 7.63 (d, j = 9 Hz, 1H, C5-H), 8.20 (s, 1H, NH; exchangeable with D_2_O); M+(*m*/*z*): 359; Anal calcd: C, 60.15; H, 5.89; N, 11.69; found: C, 60.34; H, 5.91; N, 11.88.

(*Z/E*)-1(1-(7-ethoxy-4-methylcoumarin)ethylidene)-4-benzyl-thiosemicarbazone (**VId**):

Yield = 88%; m.p. = 140–143 °C; FT-IR (ṽ max, cm^−1^): 3358 (2NH), 3100 (CH, Ar), 2980 (aliphatic CH), 1730 (C=O), 1598 (C=C, Ar), and 1184 (C-O, ether); ^1^H-NMR (300 MHz, CDCl_3_) δ (ppm): 1.41 (t, j = 6 Hz, 3H, CH_3_-CH_2_-O), 2.25 (s, 3H, -N=C-CH_3_), 2.40 (s, 3H, C-4-CH_3_), 4.16–4.21 (m, 2H, CH_3_-CH_2_-O), 4.91 (d, j = 6 Hz, 2H, CH_2_-Ph), 6.18 (s, 1H, C3-Hof coumarin), 6.92 (s, j = 9 Hz, 1H, C6-H_arom_), 7.03–7.04 (m, 5H, phenyl), 7.63 (d, j = 9 Hz,1H, C5-H_arom_), 7.84 and 8.22 (s, 2H, 2NH; exchangeable with D_2_O); M+(*m*/*z*): 409; Anal calcd: C, 64.53; H, 5.66; N, 10.26; found: C, 64.81; H, 5.74; N, 10.44.

(*Z/E*)-1(1-(7-ethoxy-4-methylcoumarin)ethylidene)-4-benzoyl-thiosemicarbazone (**VIe**):

Yield = 88%; m.p. = 220–223 °C; FT-IR (ṽ max, cm^−1^): 3468 and 3414 (2NH), 3059 (CH, Ar), 2981 (aliphatic CH), 1724 (C=O), 1597 (C=C, Ar), and 1174 (C-O, ether); ^1^H-NMR (400 MHz, CDCl_3_) δ (ppm): 1.41 (t, j = 8, 3H, CH_3_-CH_2_-O,), 2.45 (s, 3H, N=CCH_3_), 2.53 (s, 3H, C4-CH_3_), 4.21 (q, j = 8 Hz, 2H, CH_3_-CH_2_-O), 6.18 (s, 1H, C3-H of coumarin), 7.01 (d, j = 8 Hz, 1H, C6-H), 7.45 (t, t, j = 8, 2H, 3-H and 5-H _arom_), 7.56 (t, j = 8 Hz, 1H, 4-H of phenyl,), 7.69 (d, j = 8 Hz, 1H, C5-H), 7.92 (d, 2H, C2-H and C6-H of phenyl), 8.93 and 12.96 (s, 2H, 2NH; exchangeable with D_2_O); ^13^C-NMR (400 MHz, CDCl_3_) δ (ppm) = 14.60 (CH_3_-CH_2_-), 18.76 (CH_3_-), 24.10 (C=N-CH_3_), 64.81 (CH_3_-CH_2_-), 111.19–114.27 (4C of coumarin), 127.41–133.48 (7C, Ar), 150.86 (C10 of coumarin), 151.98 (-C=O of coumarin), 154.55 (C4 of coumarin), 157.42 (C_2_H_5_-O-C), 159.80 (-C=N-), 166.30 (C=O-Ph), 176.43 (-C=S); M+(*m*/*z*): 423; Anal calcd: C, 62.40; H, 5.00; N, 9.92; found: C, 62.57; H, 5.18; N, 10.04.

General procedures for the synthesis of thiazolidine-4-ones (**VII** and **VIII**): as reported [33].

The intermediates **V** or **VI** (0.005 mol) and chloroacetic acid (0.00505 mol, 0.618 g) in sodium acetate (0.00505 mol, 0.414 g) and 15 mL absolute ethanol were reacted for 8 h under reflux. After that, washing with iced cold water separated the crystalline solid that was collected and recrystallized from ethanol.

(*Z/E*)-2-{[1-(7-Ethoxy-4-methylcoumarin-8-yl)-ethylidene]-hydrazono}-3-methyl-thiazolidin-4-one (**VIIIa**):

Yield = 89%; m.p. = 245–247 °C; FT-IR (ṽ max, cm^−1^): 3040 (CH, Ar), 2985 (aliphatic CH), 1735 and1722 (2C=O), 1624 (C=N, imine), and 1600 (C=C, Ar); ^1^H-NMR (400 MHz, CDCl_3_) δ (ppm): 1.44 (t, j = 8 Hz, 3H, CH_3_-CH_2_-O), 2.39 (s, 3H, 4-CH_3_), 2.42 (s, 3H, N=C-CH_3_), 3.36 (s, 3H, 4-N-CH_3_), 3.76 (s, 2H, S-CH_2_-CO), 4.17 (q, j = 8 Hz, 2H, CH_3_-CH_2_-O), 6.16 (s, 1H, C3-Hof coumarin), 6.91 (d, j = 8 Hz, 1H, C6-H_arom_), 7.55 (d, j = 8 Hz, 1H,C5-H_arom_,); M+ (*m*/*z*): 373; Anal calcd: C, 57.89; H, 5.13; N, 11.25; found: C, 58.04; H, 5.19; N, 11.39.

(*Z/E*)-2-{[1-(7-Ethoxy-4-methylcoumarin-8-yl)-ethylidene]-hydrazono}-3-ethyl-thiazolidin-4-one (**VIIIb**):

Yield = 89%; m.p. = 177–179 °C; FT-IR (ṽ max, cm^−1^): 3086 (CH, Ar), 2978 (aliphatic CH), 1730 and1720 (2C=O), and 1624 (C=N, imine), 1600 (C=C, Ar); ^1^H-NMR (400 MHz, CDCl_3_) δ (ppm): 1.36 (t, j = 8 Hz, 3H, CH_3_-CH_2_-N-thiazolidine), 1.44 (t, j = 8 Hz, 3H, CH_3_-CH_2_-O), 2.38 (s, 3H, 4-CH_3_), 2.41 (s, 3H, N=C-CH_3_), 3.74 (s, 2H, S-CH_2_-CO), 3.94 (q, j = 8 Hz, 2H, 4-N-CH_2_-CH3), 4.11–4.18 (m, 2H, CH_3_-CH_2_-O), 6.15 (s, 1H, C3-Hof coumarin), 6.87 (d, j = 8 Hz, 1H, C6-H_arom_), 7.55 (d, j = 8 Hz, 1H, C5-H_arom_); M+(*m*/*z*): 387; Anal calcd: C, 58.90; H, 5.46; N, 10.85; found, C, 59.07; H, 5.54; N, 11.02.

(*Z/E*)-3-Allyl-2-{[1-(7-ethoxy-4-methylcoumarin-8-yl)-ethylidene]-hydrazono}-thiazolidin-4-one (**VIIIc**):

Yield = 92%; m.p. = 222–224 °C; FT-IR (ṽ max, cm^−1^): 3061 (CH, Ar), 2981 (aliphatic CH), 1737 and1726 (2C=O), 1612 (C=N, imine), and 1604 (C=C, Ar and allyl); ^1^H-NMR (400 MHz, CDCl_3_) δ (ppm): 1.44 (t, j = 8 Hz, 3H, CH_3_-CH_2_-O), 2.35 (s, 3H, 4-CH_3_), 2.42 (s, 3H, N=C-CH_3_), (s, 2H, S-CH_2_-CO), 4.13 (d, j = 8 Hz, 2H, allylic CH_2_), 4.19 (q, j = 8 Hz, 2H, CH_3_-CH_2_-O), 5.41 (d, j = 8 Hz, 2H, CH_2_-CH=CH_2_), 5.96 (m, 1H, CH_2_-CH=CH_2_), 6.16 (s, 1H, C3-Hof coumarin), 6.91 (d, j = 8 Hz, 1H, C6-H), 7.56 (d, j = 8 Hz, 1H,C5-H); ^13^C-NMR (400 MHz, CDCl_3_) δ (ppm) = 14.67 (CH_3_-CH_2_-), 18.71 (CH_3_-), 23.30 (C=N-CH_3_), 32.52 (CH_2_-Thiazolidine), 45.04 (CH_2_, Allyl), 64.62 (-CH_2_ of ethyl), 107.96–116.26 (4C of coumarin), 118.19 (-CH2 of allyl), 125.29 (C5 of coumarin), 130.26 (CH of allyl), 150.33 (C10 of coumarin), 152.25 (C4-CH_3_ of coumarin), 157.46 (C-O, ethyl), 159.28 (C=N, thiazolidine), 159.52 (C=N), 160.57 (-C=O of coumarin), 171.50 (-C=O, thiazolidine); M+(*m*/*z*): 399; Anal calcd: C, 60.13; H, 5.30; N, 10.52; found, C, 6.28; H, 5.34; N, 10.66.

(*Z/E*)-3-Benzyl-2-{[1-(7-ethoxy-4-methylcoumarin-8-yl)-ethylidene]-hydrazono}-thiazolidin-4-one (**VIIId**):

Yield = 86%; m.p. = 171–172 °C; FT-IR (ṽ max, cm^−1^): 3040 (CH, Ar), 2980 (aliphatic CH), 1724 (C=O), 1618 (C=N, imine), and 1597 (C=C, Ar); ^1^H-NMR (400 MHz, CDCl_3_) δ (ppm): 1.44 (t, j = 8 Hz, 3H, CH_3_-CH_2_-O), 2.39 (s, 3H, 4-CH_3_), 2.43 (s, 3H, N=C-CH_3_), 3.77 (s, 2H, S-CH_2_-C-O), 4.56 (q, j = 8 Hz, 2H, CH_3_-CH_2_-O), 5.05 (s, 2H, Benzyl CH_2_), 6.12 (s, 1H, C3- of coumarin), 6.88 (d, j = 8 Hz, 1H, C6-H_arom_), 6.89–7.39 (m, 5H, Ar), 7.55 (d, j = 8 Hz, 1H, C5-H); M+(*m*/*z*): 449; Anal calcd: C, 64.13; H, 5.16; N, 9.35; found: C, 64.25; H, 5.23; N, 9.44.

(*Z/E*)-3-Benzoyl-2-{[1-(7-ethoxy-4-methylcoumarin-8-yl)-ethylidene]-hydrazono}-thiazolidin-4-one (**VIIIe**):

Yield = 50%; m.p. = 242–244 °C; FT-IR (ṽ max, cm^−1^): 3064 (CH, Ar), 2981 (aliphatic CH), 1716 and 1670 (2C=O), 1624 (C=N, imine), and 1597 (C=C, Ar); ^1^H-NMR (400 MHz, CDCl_3_) δ (ppm): 1.41 (t, j = 8 Hz, 3H, CH_3_-CH_2_-O), 2.35 (s, 3H, 4-CH_3_), 2.46 (s, 3H, N=C-CH_3_), 3.86 (s, 2H, S-CH_2_-CO), 4.17 (q, j = 8 Hz, 2H, CH_3_-CH_2_-O), 6.16 (s, 1H, C3-H of coumarin), 6.93 (d, j = 8 Hz, 1H, C6-H_arom_), 7.38–7.62 (m, 3H, C3, C4, C5-H_arom_ of phenyl), 7.90 (d, j = 8 Hz, 2H, C2, C6-H_arom_ of phenyl), 7.83 (d, 1H, C5-H_arom_); M+(*m*/*z*): 463; Anal calcd: C, 62.19; H, 4.57; N, 9.07; found, C, 62.37; H, 4.61; N, 9.13.

### 3.2. Antitumor Activity

#### 3.2.1. Cytotoxicity Assay

Cell culture:

MCF-7 breast cancer cell line (ATCC^®^ HTB-22™) plus non-tumorigenic epithelial cell line (MCF 10, ATCC^®^ CRL-10317™) were supplied from VACSERA (Cairo, Egypt) and cultured in Dulbecco’s Modified Eagle Medium (Invitrogen-Life Technologies, Carlsbad, CA, USA) with 1% antibiotic solution (streptomycin–penicillin) plus 10% fetal bovine serum (Hyclone) in a 5% (*v*/*v*) humidified CO_2_ incubator at 37 °C.

MTT assay:

IC_50_ of each of the tested compounds was studied by MTT assay where cells were treated with trypsin, counted, and then plated in sterile microtiter plates (density: 1.2–1.8 × 10^4^ cells/well). Firstly, cells were kept in a humidified atmosphere (37 °C, 24 h), and then incubated with serial concentrations of the tested compounds. After 48 h, the medium was aspirated, and then cells were incubated with 5% MTT solution (M-5655; Sigma Aldrich, St. Louis, MO USA) (200 µL/well, 2 h), allowing the dye to metabolize into the colored insoluble formazan complex that was then dissolved in the appropriate solubilization solution (M-8910) (200 µL/well), for 30 min with gentle mixing at room temperature. The UV absorbance was measured using a microplate reader (570 nm), and cell viability was determined with respect to untreated control cells. The cytotoxic potencies of synthesized derivatives were expressed as IC_50_ value, which represents the concentration of tested compound capable of inducing 50% inhibition in cell proliferation. The values were means ± sd; *n* = 3.

#### 3.2.2. PI3K and Akt Enzyme Inhibition Assays

The in vitro inhibition of PI3K and Akt kinase activities by **VIIb** was assessed using a PI3Kα (p110α/p85) assay kit (Catalog #79781; BPS Bioscience, Inc, San Diego, CA, USA), PI3Kγ (p110γ/PIK3R5) assay kit (Catalog #79803; BPS Bioscience, Inc, San Diego, CA, USA), and Akt Kinase Activity assay kit (ab139436; Abcam, Cambridge, UK) following the manufacturer’s instructions as described previously [58,59]. The results were expressed as IC_50_ values using dose–response curves and linear regression equations. LY294002 compound was taken as the reference compound. The values were means ± sd; *n* = 3.

#### 3.2.3. Cell Cycle Analysis and Apoptosis Induction

Cell cycle analysis and apoptosis rates following **VIIb** treatment were investigated using a Propidium Iodide Flow Cytometry Kit (ab139418; Abcam, Cambridge, UK) [60] and Annexin V-FITC apoptosis kit (Catalog: K101-25; BioVision Research Products, San Francisco, CA, USA) [61], respectively, following the manufacturer’s instructions as stated by a previous study [17].

#### 3.2.4. Determination of the Cleaved Caspase-9 Level

The level of cleaved caspase-9 was assessed in both untreated/control MCF-7 cells and following **VIIb** treatment using DRG^®^ Caspase-9 (human) ELISA (EIA-4860), following the manufacturer’s instructions (DRG International Inc., Springfield, NJ, USA). The data were means ± sd; *n* = 3.

#### 3.2.5. Western Blot

Western blot analysis was conducted using the following primary antibodies: anti-p-Akt (1:5000; ab81283), anti-Cyclin D1 (1:1000; ab226977), anti-p-PI3K (1:1000; ab278545), and anti-beta-actin (1:1000; ab8227). Firstly, **VIIb**-treated and untreated cells were rinsed with PBS, and then cold lysis buffer was added to induce cell lysis. After centrifugation, the supernatants were collected, and harvested proteins were quantified by Bradford assay and then resolved on SDS-PAGE followed by electroblotting onto polyvinylidene fluoride membranes. Later, membrane blocking was conducted using 5% skimmed milk in 0.1% Tween-20 in PBS (PBST). Then membranes were incubated with the aforementioned primary antibodies at 4 °C. Following 12-h incubation, the membranes were soaked in PBST thrice followed by 1-h incubation with appropriate secondary antibodies. Antibodies were supplied from Abcam (Cambridge, UK). The signals were visualized with a chemiluminescence ECL kit (Perkin Elmer, Waltham, MA, USA) following the manufacturer’s instructions, and images were obtained using a Biorad Imager.

#### 3.2.6. Molecular Simulation Studies

Molecular docking of the two target compounds into the PI3K protein (PDB code 4L23) and Akt protein (PDB code 3O96) was performed using the MOE software package. Initially, the downloaded protein was prepared, and water molecules were removed followed by the minimization step. The standard settings were kept in the docking steps. The 2D interaction diagrams for the best 10 poses were studied, and the highest-scoring binding poses were selected and compared to the reference ligands.

### 3.3. Statistical Analysis

Unpaired *t*-tests (GraphPad Prism v7.00) were performed to investigate the significance levels between tested Compound **VIIb** and control samples or reference compounds. *p* < 0.05 was considered significant.

## 4. Conclusions

By adopting the pharmacophore hybridization approach, new series of 7-hydroxyl-4-methylcoumarin and their 7-ethoxy analogs bearing thiosemicarbazone (**V–VI**) or thiazolidin-4-one moiety (**VII** and **VIII**) were designed, prepared, and then their in vitro cytotoxicity against MCF-7 cells examined by MTT assay. Nine compounds, namely **Va**, **VIa**, **VIc**, **VId**, **VIf**, **VIIb**, **VIIIa VIIIc**, and **VIIIf**, demonstrated significant cytotoxicity; thus, they are considered promising antiproliferative agents against MCF-7 cells. Overall, the present study exemplified that one of these derivatives, **VIIb**, induced significant cytotoxicity at a low concentration of 1.03 ± 0.05 µM. Further investigations were conducted to unravel the mechanistic details of this observation. Mechanistically, **VIIb** exerted its effect via dual inhibition of PI3K/Akt kinase activity, as manifested by the results of enzyme inhibition assay, and was further confirmed by Western blot results. Additionally, **VIIb** treatment induced S-phase cell cycle arrest alongside induction of caspase-9 mediated apoptosis. Further, Western blot results demonstrated potential Compound **VIIb** modulation of anti-apoptotic Cyclin D1, as evidenced by its decreased protein expression. Eventually, molecular docking illustrated the binding patterns of this compound with the targeted enzymes PI3K and Akt-1. These newly designed and synthesized coumarin hybrids are excellent candidates for further investigation and optimization targeting signal transduction pathways in the treatment of cancer. In particular, the currently observed antitumor efficacy of the novel coumarin derivative **VIIb** in MCF-7 cells suggests the potential to evolve as a promising anti-cancer compound via dual inhibition of the PI3K/Akt axis.

## Figures and Tables

**Table 1:** The values of IC_50_ (µM) of the tested compounds in MCF-7 cells.

Compound No.	R	R1	IC_50_ (µM)
**5-FU**	-	-	27.81 ± 1.41
**Va**	H	Ph-CH_2_	5.13 ± 0.28 ***
**Vb**	H	Ph-C=O	47.32 ± 2.47
**VIa**	C_2_H_5_	CH_3_	11.13 ± 0.58 ***
**VIb**	C_2_H_5_	C_2_H_5_	38.70 ± 2.09
**VIc**	C_2_H_5_	CH_2_=CH-CH_2_	2.72 ± 0.13 ***
**VId**	C_2_H_5_	Ph-CH_2_	2.61 ± 0.14 ***
**VIe**	C_2_H_5_	Ph-C=O	43.05 ± 2.25
**VIf**	C_2_H_5_	4-OCH_3_-Ph	1.21 ± 0.06 ***
**VIIa**	H	Ph-CH_2_	54.80 ± 2.86
**VIIb**	H	Ph-C=O	1.03 ± 0.05 ***
**VIIIa**	C_2_H_5_	CH_3_	20.27 ± 1.06 **
**VIIIb**	C_2_H_5_	C_2_H_5_	57.28 ± 2.99
**VIIIc**	C_2_H_5_	CH2=CH-CH_2_	4.95 ± 0.26 ***
**VIIId**	C_2_H_5_	Ph-CH_2_	79.93 ± 4.18
**VIIIe**	C_2_H_5_	Ph-C=O	26.41 ± 1.38 ^ns^
**VIIIf**	C_2_H_5_	4-OCH_3_-Ph	11.80 ± 0.62 ***

IC_50_ values are means ± sd; *n* = 3. *p*-value using independent *t*-test for tested compounds vs. Compound 5-Fluorouracil (5-FU). *** *p* ≤ 0.001, ** *p* ≤ 0.01. ^ns^ Non-statistically significant *p* > 0.05.

**Table 2:** IC_50_ values (µM) of the most cytotoxic compounds (**VIf** and **VIIb**) against MCF-10A cell line and their selectivity indices.

Compound	IC_50_ (µM) MCF-10 ^a^	IC_50_ (µM) MCF-7	Selectivity Index MCF-10/MCF-7
**VIf**	20.11 ± 1.05	1.21 ± 0.06	16.61
**VIIb**	9.52 ± 0.60	1.03 ± 0.05	9.24
**5-FU**	36.22 ± 1.89	27.81 ± 1.41	1.30

^a^ IC_50_ values are means ± sd; *n* = 3.

**Table 3:** IC_50_ values (µM) for the inhibition of PI3K-α, PI3K-γ, and Akt-1 enzymes following Compound **VIIb** treatment.

Compounds	IC_50_ (µM)		
	PI3K-α Isoform	PI3K-γ Isoform	Akt-1 Isoform
**VIIb**	3.70 ± 0.19 ***	34.70 ± 1.88	2.93 ± 0.15 *
**LY294002**	8.85 ± 0.46	11.5 ± 0.62	3.53 ± 0.18

Values are means ± sd; *n* = 3. *p*-value using independent *t*-test for **VIIb** vs. LY294002 reference compound. *** *p*≤ 0.001, * *p* ≤ 0.05.

**Table 4:** Cell cycle analysis following 48-h treatment with Compound **VIIb**.

Cells	DNA Content %			
	G0/G1	S	G2/M	Pre G1
**VIIb**-treated cells	48.39	46.02	5.59	35.25
Control untreated cells	53.71	36.58	9.71	1.55

**Table 5:** Percentage of apoptotic cells after Compound **VIIb** treatment.

Cells	Apoptosis %			Necrosis%
	Total	Early	Late	
**VIIb**-treated cells	35.25	2.51	21.05	11.69
Control untreated cells	1.55	0.48	0.17	0.90

**Table 6:** Effect of **VIIb** treatment on caspase-9 level in MCF-7 cell line.

Cells	Caspase-9 Level (ng/mL)	Fold
**VIIb**-treated cells	21.61 ± 0.14 ***	7.96
Control untreated cells	2.712 ± 0.09	1

Values are means ± sd; *n* = 3. *p*-value using independent *t*-test for **VIIb** vs. control untreated cells. *** *p* ≤ 0.001.

**Table 7:** The docking scores and the main H-bonds observed of the most active compounds against PI3K active site.

Compound	S-Score	Interaction Types and Residues
**1 X6K**	−2.2807	H-bonding, Lys802
**2 VIIb**	−3.7521	H-bonding, Tyr836
**3 VIf**	−3.0518	H-bonding, Lys802

**Table 8:** The docking scores and the main interactions observed of the most active compounds against Akt active site.

Compound	S-Score	Interaction Types and Residues
**1 IQO**	−5.0984	Hydrophobic aromatic, Trp80, Arg273; H-bonding, Asn54.
**2 VIIb**	−2.6564	H-bonding, Trp80
**3 VIf**	−4.3739	H-bonding, Trp80

## Data Availability

Data is contained within the article.
